# Degradation of extracellular polymeric substances shapes microbial community diversity

**DOI:** 10.1371/journal.pbio.3003287

**Published:** 2025-07-29

**Authors:** Sammy Pontrelli, Kian Bigovic Villi, Andreas Sichert, Julian Trouillon, Adriano Rutz, Zachary C. Landry, Simon H. Rüdisser, Roman Stocker, Uwe Sauer

**Affiliations:** 1 Institute of Molecular Systems Biology, ETH Zürich, Zürich, Switzerland; 2 Department of Biology, KU Leuven, Leuven, Belgium; 3 VIB-KU Leuven Center for Microbiology, Leuven, Belgium; 4 Department of Civil, Environmental and Geomatic Engineering, Institute of Environmental Engineering, ETH Zurich, Zürich, Switzerland; 5 Department of Marine and Environmental Biology, University of Southern California, Los Angeles, California, United States of America; 6 Department of Biology, ETH Zürich, Zürich, Switzerland; Fred Hutchinson Cancer Research Center, UNITED STATES OF AMERICA

## Abstract

Metabolic cross-feeding networks are central to shaping microbial community dynamics in environments ranging from the rhizosphere, gut, and marine carbon cycling. Yet cross-feeding has predominantly been viewed by examining exchanged small metabolites. In contrast, the role of extracellular polymeric substance (EPS)—a complex mixture of proteins, polysaccharides, DNA, and humic-like compounds—in cross-feeding remains poorly understood, mainly due to technical challenges in measuring their secretion relative to small metabolites. Using chitin-degrading microbes as a model system, we used a bicarbonate-buffered bioreactor coupled with elemental analysis, which allowed us to quantify both EPS and small metabolite secretion. This revealed that ~25% of carbon exuded by a chitin degrader is in the form of EPS. EPS was produced at similar levels across marine chitin-degrading isolates and seawater communities, underscoring its importance relative to small metabolites. Notably, different sources of EPS were found to select for distinct and diverse microbial communities. Combining in vitro enzyme assays and untargeted metabolomics, we show that EPS undergoes sequential degradation—from large oligomers to smaller, broadly accessible monomers. This sequential breakdown creates a temporal succession of metabolic niches, potentially fueling a shift from specialist species degrading complex substrates to a more diverse community of generalists using simpler monomers. By identifying EPS as a major and dynamic contributor to cross-feeding networks, our findings reveal a hidden layer of complexity in how microbial communities assemble and function across ecosystems.

## Abstract 2

Cross-feeding of small metabolites is known to drive carbon exchange among microbial species, but the role of extracellular polymeric substances (EPS) has been poorly understood. This study shows that EPS, formed during chitin degradation, drives microbial diversity by acting as a major, sequentially degraded, carbon source.

## Introduction

Microbial communities are essential for biogeochemical cycles and the health of humans, animals, and plants [1,2]. A pressing goal is to identify the key factors for controlling community structure and population dynamics. One major focus is how metabolites are exchanged in cross-feeding networks, where a plethora of studies have demonstrated their pivotal role. For example, how complex metabolite pools are partitioned among metabolically diverse species [3–5], how temporal shifts in metabolite pools select for distinct subpopulations [3,6,7], and how phage or stress-induced release of intracellular metabolites fuel surrounding species [8,9]. These findings have helped distill the complexity of microbial communities into more tractable concepts. However, most studies have focused on carbon cross-feeding via low-molecular-weight (LMW) metabolites [3,6,7,10,11]—monosaccharides, organic acids, amino acids, and vitamins—which are core building blocks that can be rapidly exuded and consumed between broad sets of species.

Extracellular polymeric substances (EPSs) are another potential source of cross-fed compounds [12–16]. EPS includes polysaccharides, nucleic acids, proteins, and humic-like substances [13]. Due to its size and complexity, high-molecular-weight EPS is often overlooked in cross-feeding studies, as it is not captured by metabolomics that resolve only LMW compounds in microbial exudates. However, EPS is ubiquitous across microbial ecosystems and serves diverse roles. For example, biofilms are largely composed of chemically diverse polysaccharides [17–19]; secreted proteins degrade external polymers [20,21]; and humic-like substances form during microbial breakdown of organic matter [22]. Given EPS’s widespread presence across ecosystems, and the ability of specialized microbes to degrade specific EPS components [13,16], it may play a universal role in shaping microbial community structures. Indeed, EPS composition has been shown to select distinct communities across diverse ecosystems [16,23,24]. Yet, key questions remain: how does EPS contribute to carbon exchange relative to LMW metabolites, which EPS components drive cross-feeding, and how does EPS shape functional community structure? These gaps point to an underappreciated factor shaping microbial population dynamics across diverse habitats.

This study investigates how EPS mediates carbon exchange and shapes population dynamics in a chitin-degrading microbial community. Chitin, an insoluble polysaccharide found in crustacean and copepod shells, is one of the most abundant polysaccharides in the ocean and plays a significant role in the global carbon cycle [25]. Our previous studies have demonstrated the influence of metabolic crossfeeding on chitin-degrading communities, specifically that released LMW metabolites by specialized chitin degraders have a cascading influence on the population dynamics of subsequently colonizing species [6,26,27]. While these studies emphasized the importance of LMW metabolites, EPS has the potential to play a major role in carbon cross-feeding and community dynamics, as chitin-degrading microbes inherently release EPS [28–30]—either as chitinases and accessory proteins to hydrolyze chitin or as biofilms to form cell aggregates or adhere to particle surfaces.

Chitin-degrading communities offer a unique opportunity to quantify the relative contributions of directly secreted EPS and LMW metabolites to carbon flux, as all carbon that fuels non-degrading microbes and cross-feeding networks originates from specialized degraders which release extracellular compounds. Unlike communities grown in complex media, this allows us to directly quantify and compare the formation of EPS and LMW metabolites during chitin breakdown. In this work, we used a bicarbonate-buffered bioreactor with elemental analysis to quantify EPS and LMW metabolites in microbial exudates, providing a basis to assess their relative contributions to carbon cross-feeding. We also applied enzyme assays and metabolomics to track EPS degradation over time. Our findings show that EPS contributes a comparable amount of cross-fed carbon as LMW metabolites and is degraded in multiple enzymatic steps, generating temporal metabolite niches that can sustain metabolically diverse communities. This positions EPS as a key ecological factor shaping microbial community composition and function and highlights its overlooked role relative to LMW metabolites in driving carbon cross-feeding.

### EPS yields and compositions during chitin degradation

**Fig 1:**
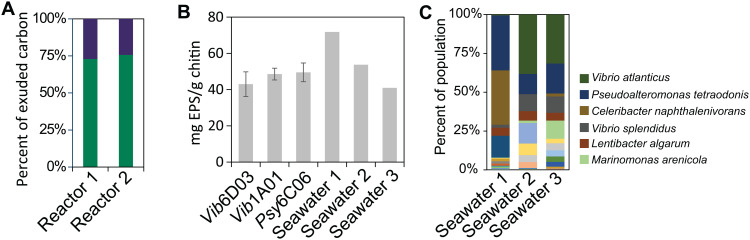
EPS yields from chitin. **A)** The fraction of carbon found in either EPS (blue) or LMW metabolites (green), relative to the total exuded carbon (EPS + LMW), based on elemental analysis of two replicate bioreactors. **B)** EPS yields in early stationary phase after growth on 2 g/L of chitin as the sole carbon source. The three species are known chitin degraders and the three seawater inocula. Error bars represent the standard deviation of the mean from three biological replicates. **C)** 16S sequences of the three seawater communities at the time of EPS harvesting, showing the average top 6 most abundant species. Raw data for all plots can be found in S1 Data.

To assess the potential role of EPS in carbon flux between degraders and non-degraders, we quantified the proportion of chitin-derived carbon allocated to EPS (larger than 3 kDa), LMW metabolites (below 3 kDa), and biomass in the model chitin-degrading bacterium *Vib*1A01 [9,31,32]. Upon reaching early stationary phase on chitin as the sole carbon source, we collected biomass, separated EPS from LMW fractions using a 3 kDa filter, and measured the carbon content in each fraction—expressed as a percentage of the total carbon from the chitin substrate—using elemental analysis. The use of a bicarbonate-buffered bioreactor allowed carbon quantification of the LMW fraction via elemental analysis, by enabling removal of dissolved inorganic carbon prior to measurement. Of the 2 g/L chitin that were completely consumed (with residual concentrations below the detection limit of 62 mg/L), 33%–36% of the input chitin carbon was recovered in biomass, 4.1%–4.5% in EPS, and 11%–14% in LMW metabolites, while the remaining unaccounted 45%–52% were deemed lost as CO_2_ in respiration. This corresponds to EPS making up approximately one-quarter of the total exuded carbon (EPS + LMW) (Fig 1A).

We next asked whether EPS production was consistent across other degraders and microbial communities. To do this, we measured EPS yield as the mass of dried EPS per gram of chitin consumed. EPS yields from *Vib*1A01 (~45 mg/g chitin) were similar to those of two other chitin-degrading isolates (Fig 1B). When chitin cultures were inoculated with non-sterilized seawater, we observed comparable EPS yields of 4–7%, independent of variations in community composition as determined through 16S sequencing (Fig 1C, Bray–Curtis dissimilarity: 0.78 and 0.69 for communities 1 versus 2 and 3, and 0.31 for 2 versus 3). This consistency in EPS yield across strains and natural communities suggests that EPS production is a robust and general feature of chitin degradation.

**Fig 2:**
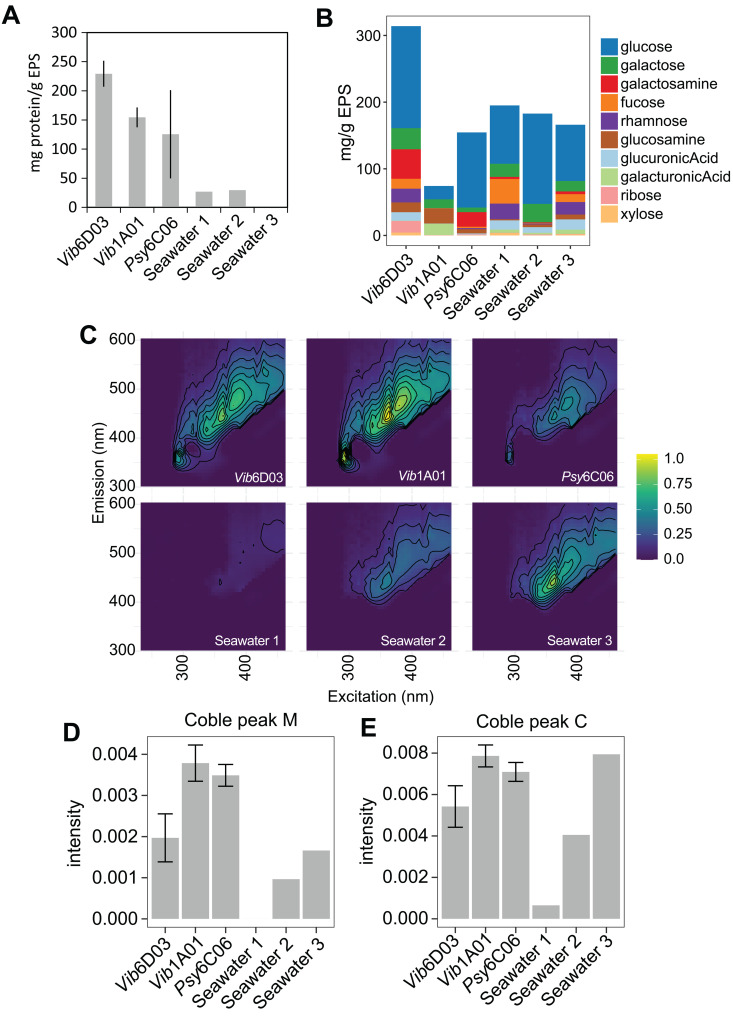
Composition of EPS. **A)** Mass fraction of proteins in EPS from different sources. **B)** Mass fraction of monosaccharides in hydrolysable polysaccharides in EPS from different sources (S1 Table). **C)** Excitation emission matrices of each EPS at 1 g/L. Raw data are in S3 Data. **D)** Coble peak M (Ex/Em 290-310/370-410) and peak C (Ex/Em 320-360/420-460) in the excitation emission matrices. Error bars represent standard deviation of the mean of three biological replicates. Raw data for panels ABDE can be found in S2 Data, and panel C in S3 Data.

Next, we investigated the molecular composition of the six EPS fractions—derived from three degraders and three seawater communities—because EPS is a heterogeneous mix of macromolecules [13]. In degrader monocultures, proteins, which are naturally secreted for chitin degradation, comprised 13%–23% (g/g) of the EPS. In seawater communities, proteins accounted for less than 3% or were undetectable (Fig 2A), suggesting that proteins may have been consumed by non-degrading microbes. Polysaccharides, which play critical roles in cell adhesion, bacterial aggregation, and biofilm formation, were subjected to acid hydrolysis, and the resulting monosaccharides were quantified using liquid chromatography-mass spectrometry (S1 Table). The polysaccharide content varied widely, representing 9%–36% of the total carbon in the six EPS samples (Fig 2B). Glucose emerged as the most abundant monosaccharide in five of the samples, averaging 55% of the monomers by weight. DNA was found to be at negligible concentrations, roughly 0.17% (g/g) of *Vib*1A01 EPS. Overall, proteins and hydrolysable polysaccharides constituted 22%–59% (g/g) of the exuded EPS.

High-molecular-weight humic-like substances, which form naturally during bacterial degradation of organic matter [33], can be important components of EPS that may also support specific microbes [34]. Their complex structures complicate quantification; however, fluorescent signatures, such as the Coble M peak (Ex 290–310 nm, Em 370–410 nm) for marine and Coble C peak (Ex 320–360 nm, Em 420–460 nm) for visible humic-like substances, can revealed their presence [35]. We obtained excitation–emission matrices and detected both C and M peaks in the EPS of all three degraders (Fig 2C, 2D, and 2E). In two of the three seawater samples, however, the C and M peaks were much lower than in the monocultures. These variations in humic-like substances, along with differences in protein and polysaccharide content, illustrate diverse EPS composition across different sources of EPS.

### Influence of EPS composition of microbial community structure

Given the variable EPS composition, we hypothesized that their unique components would select for distinct microbial communities of heterotrophic bacteria. To test this hypothesis, we cultured microbes naturally found in seawater using EPS as the sole carbon source and analyzed the community composition through 16S sequencing. To prevent the formation of entirely distinct community assemblages from the same dilute seawater inoculum (as shown in Fig 1B), we initially prepared the inoculum by inoculating a minimal medium supplemented with five different carbon sources with seawater. This approach generated a dense and diverse culture, which was then used to seed the various EPS media. As a result, any observed changes in community composition can be attributed specifically to the different EPS substrates rather than to random inoculum variations.

**Fig 3:**
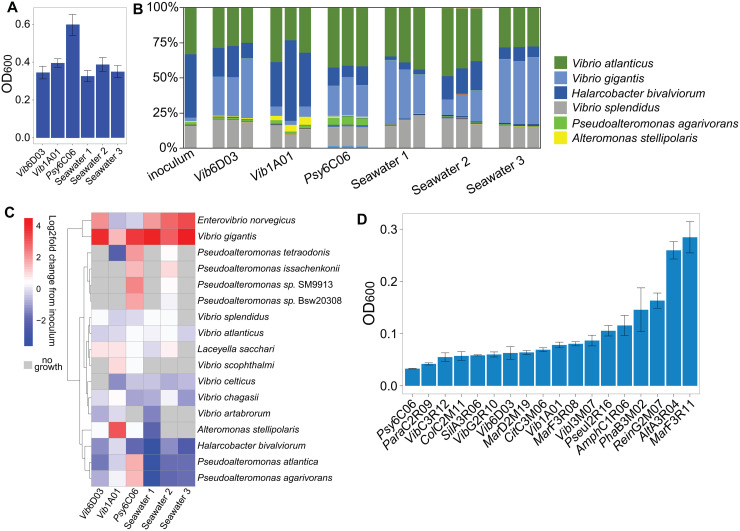
Growth selection by EPS as a carbon source. **A)** Optical density 48 h after inoculation with seawater on 1 g/L EPS as a sole carbon source, derived from the indicated chitin-degrading cultures. **B)** 16S sequencing of seawater communities after growth on each EPS medium. **C)** Heatmap showing Log_2_ fold change in abundance of species in the EPS communities compared to the inoculum, where species not found in the EPS communities are shown in gray. Species are clustered using Ward’s method. **D)** Growth of individual bacterial isolates on 1 g/L of EPS from *Vib*1A01 as a sole carbon source at 72 hrs. All error bars represent standard deviation of three biological replicates. Raw data for all plots can be found in S4 Data.

After 48 h, robust growth was observed on all EPS substrates (Fig 3A). While communities shared some abundant taxa, each assembled with distinct population structures, uniquely enriched with specific degraders compared to the inoculum (Fig 3B and 3C). Notably, *Vibrio gigantis*, a marine polysaccharide degrader [36], increased 7- to 22-fold in five out of the six EPS conditions when compared to the inoculum. In the sixth condition, *Alteromonas stellipolaris,* a representative of the *Alteromonas* genus known for its ability to degrade algal polysaccharides [37] and polycyclic aromatic hydrocarbons [38], increased nearly 9-fold. Moreover, six *Pseudoalteromonas* species, known for degrading marine polysaccharides and proteins [39,40], increased up to 5-fold exclusively in *Psy*6C06 EPS, demonstrating EPS specificity in selecting distinct EPS-degrading microbes. To further demonstrate that EPS degradation is a specialized trait, we screened the growth of 18 bacteria from a model chitin-degrading community [6] on the EPS of *Vib*1A01 as the sole carbon source (Table 1). While most species displayed some growth, *Alt*A3R04 and *Mar*F3R11 grew to the highest OD_600_ (Fig 3D). Overall, these results show that different EPS types select for specific degraders, which may subsequently influence community structure.

**Table 1:** Species used in this study and NCBI accession numbers for genomic sequences. The functional guild describes whether species have the ability to degrade chitin and grow on it as a sole carbon source.

Species	Taxonomic ID	Functional Guild	NCBI BioProject	NCBI BioSample
AltA3R04	*Alteromonas sp.*	Non-degrader	PRJNA478695	SAMN19350919
AmphC1R06	A*mphritea* sp.	Non-degrader	PRJNA478695	SAMN19350932
CitC3M06	*Citricella* sp.	Non-degrader	PRJNA478695	SAMN19350936
ColC2M11	*Colwellia psychrerythraea*	Non-degrader	PRJNA478695	SAMN19350933
MarD2M19	*Marinobacter sp.*	Non-degrader	PRJNA478695	SAMN19350941
MarF3R08	*Marinobacter* sp.	Non-degrader	PRJNA478695	SAMN19350954
MarF3R11	*Marinobacter* sp.	Non-degrader	PRJNA478695	SAMN09522136
ParaC2R09	*Paracoccus kamogawaensis*	Non-degrader	PRJNA478695	SAMN19350935
PhaB3M02	*Phaeobacte*r sp.	Non-degrader	PRJNA478695	SAMN09522130
PseuI2R16	Pseudoalteromonadaceae	Non-degrader	PRJNA478695	SAMN19350960
Psy6C06	*Psychromonas* sp.	Degrader	PRJNA414740	SAMN08130274
ReinG2M07	*Reinekea* sp.	Non-degrader	PRJNA478695	SAMN19350955
SilA3R06	*Silicibacter* sp.	Non-degrader	PRJNA478695	SAMN19350920
Vib1A01	*Vibrio splendidus*	Degrader	PRJNA414740	SAMN07809270
Vib6D03	*Vibrio penaeicida*	Degrader	PRJNA414740	SAMN08130373
VibC3R12	*Vibrio* sp.	Non-degrader	PRJNA478695	SAMN09522132
VibG2R10	*Vibrio* sp.	Degrader	PRJNA478695	SAMN19350956
VibI3M07	*Vibrio* sp.	Degrader	PRJNA478695	SAMN19350961

### Stepwise enzymatic degradation of EPS

While EPS degradation appears to rely primarily on degrading microbes, the diversity and presence of certain species across all EPS conditions (Fig 3B and 3C) suggest that non-degrading microbes are also supported. One possibility is that these species obtain nutrients by exploiting the extracellular breakdown of EPS into monomer or oligomers without themselves releasing enzymes, similar to exploiters in other polysaccharide-degrading communities [6,41,42]. To assess the range of EPS degradation products that may fuel these species, we purified EPS from the three chitin degraders after they were grown on colloidal chitin and subsequently used it as a sole carbon source for a seawater community. Once the cultures reached early stationary phase, we removed cells by centrifugation and concentrated secreted proteins along with any remaining EPS. This concentrated enzyme mixture was then incubated in vitro with fresh EPS, and aliquots were withdrawn over a 24 h period to monitor the formation of degradation products using untargeted liquid chromatography quadrupole time of flight mass spectrometry (LC–QTOF–MS) mass spectrometry-based metabolomics.

**Fig 4:**
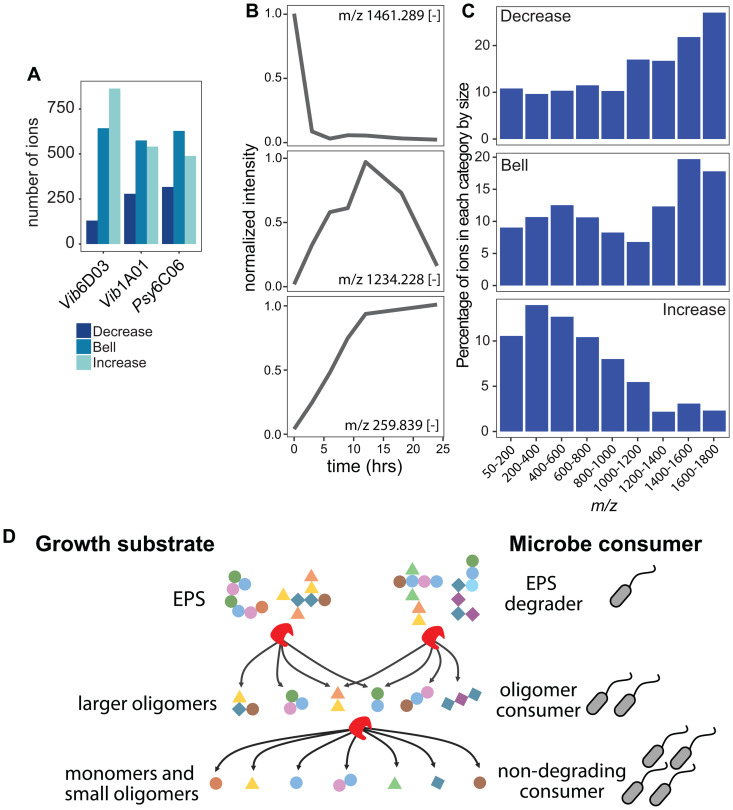
Size and temporal dynamics of EPS digestion. **A)** Number of ions in each enzyme digest falling in the decreased, bell-shaped, or increased categories. **B)** Example ions from each temporal category, ions are measured in negative mode. **C)** Percentage of all detected ions in different size ranges that fall into the three temporal categories. **D)** Graphical illustration of how EPS is enzymatically degraded in multiple steps into smaller fragments, fueling the growth of non-degrading species: EPS degraders break down EPS into larger oligomers for oligomer consumers; further degradation into monomers and small oligomers supports non-degrading consumers. Data for panels AC can be found in S5 Data. Data for panel B can be found in S7 Data.

A total of 2,820 ions exhibited one of the three distinct temporal profiles in at least one enzyme digest (Fig 4A, 4B). First, 492 ions decreased steadily in intensity, consistent with enzymatic breakdown. Second, 1,297 ions showed a bell-shaped profile, indicative of intermediate oligomers that accumulate transiently before further degradation. Third, 1,226 ions increased over time, reflecting the accumulation of terminal digestion products. Analyzing the size distribution (Fig 4C) revealed that both decreasing and bell-shaped ions predominantly comprise high-mass ions (1,600–1,800 *m/z*), whereas increasing ions were primarily of low mass (200–400 *m/z*). If bell-shaped ions are formed from degradation of the EPS substrate, we would presume that decreasing ions should be larger than bell-shaped ions. Their similar mass distributions likely result from the 1,800 *m/z* mass detection limit of our metabolomics method. Since the EPS substrate—retained by a 3 kDa molecular weight cutoff—is larger than this range, monitoring the degradation of EPS polymers and formation of very large oligomers cannot be fully captured. Despite this limitation, the observed transition from high-mass to low-mass ions illustrates a stepwise degradation in which large EPS polymers are enzymatically cleaved into transient oligomers and ultimately into small oligo- and monomers that cannot be further broken down.

If EPS is progressively degraded into simpler oligo- and monomers, this process may have ecological implications by enabling diverse, non-degrading community members to access these EPS-derived monomers. To investigate whether common, broadly accessible metabolites are produced across different enzyme digests, we analyzed ions that increased over time. We hypothesized that smaller ions would be more frequently shared across enzyme digests, while larger intermediates would be more digest-specific due to their greater structural diversity. Among ions >1,400 m/z, only 15% were detected in more than one digest, whereas over 50% of ions in the 50–200 m/z range were shared across digests (S6 Data). These findings support the hypothesis that early degradation produces digest-specific oligomers, while further hydrolysis yields more commonly produced mono- and oligomers that could be scavenged by non-degrading community members (Fig 4D).

**Table:** 

m/z	retention_time	Degrader	hours	mode	annotation	intensity

**Fig 5:**
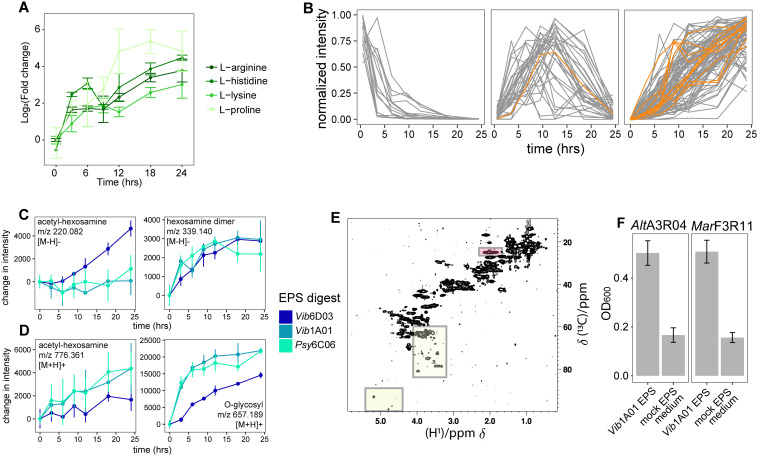
Release of amino acids and monosaccharides from EPS. A) The four amino acids showing the highest log2 fold-change in intensity (relative to time 0) generated during *Vib*6D03 EPS digestion. **B)** Temporal trajectories of annotated peptides with four or fewer amino acids formed during the enzymatic digestion of *Vib*6D03 EPS, with ions in orange annotated as peptides or amino acids based on MS^2^-based CANOPUS predictions. Intensity change of compounds in enzyme digests relative to time 0 for **C)** acetyl-hexosamine and hexosamine dimers, annotated by exact mass, and **D)** ions corresponding to acetyl-hexosamine and O-glycosyl compound classes, annotated by CANOPUS. **E)** ^1^H-^13^C HSQC NMR spectrum of EPS. The spectral regions of the carbohydrate resonances are highlighted by yellow boxes. The resonance at the chemical shift characteristic of an N-acetyl group is highlighted in red. The signals outside of these regions indicate the presence of amino acids, peptides, or small proteins. **F)** Growth of EPS-degrading microbes on 1 g/L of *Vib*1A01 enzyme digest and mock EPS medium containing proteins and monosaccharides to mimic those found in *Vib*1A01 EPS. Error bars represent standard deviation of the mean of three biological replicates. Raw data for panels AC and F can be found in S10 Data. Data for panel B can be found in S7 Data.

To identify specific degradation products, we putatively annotated 279 of the 2,820 detected ions based on their exact masses using the BioCyc database [43] (S9 Data) and used CANOPUS [44] to classify them into more detailed compound classes ions for which MS^2^ spectra were acquired (S9 Data). Consistent with proteins accounting for 13%–23% of EPS (Fig 2A), the concentrations of the 17 detectable amino acids increased by at least 2-fold in one or more digests. In particular, in the digest of *Vib*6D03’s EPS, which was found to have the highest concentration of protein (Fig 2A), we detected 15 amino acids (Fig 5A). We further annotated peptides by examining the compound classes related to their fragmentation spectra or by comparing the exact masses of detected ions to those of peptides containing four or fewer amino acids. These peptides exhibited three distinct temporal patterns: increasing, decreasing, or bell-shaped (Fig 5B). This supports the conclusion that EPS proteins are sequentially degraded into peptides, which are ultimately broken down into amino acids that provide nutrients for non-degrading microbes. Additionally, we observed accumulation of putatively annotated acetyl-hexosamine and aminohexose dimers in the digests (Fig 5C), along with acetyl-hexosamine and O-glycosyl compound classes (Fig 5D), likely arising from the degradation of EPS polysaccharides. Consistently, NMR analysis of undigested *Vib*1A01 EPS confirmed strong peaks for acetylated sugars and carbohydrates (Fig 5E), as well as for proteins. Akin to the amino acids, hexosamines and acetyl-hexosamines can support the growth of both degraders and non-degrading microbes once the polysaccharides are degraded.

The metabolomics data show that proteins and polysaccharides could potentially feed EPS-degrading communities. To investigate whether these two polymers are primarily responsible for observed growth, or if other EPS components also contribute, we designed a medium that mimics 1 g/L of *Vib*1A01 EPS. This medium contains 150 mg/mL of protein, in the form of bovine serum albumin, and the approximate composition of all monosaccharides present at concentrations greater than 1 µM from the polysaccharide fraction (S1 Table). The EPS-degrading isolates *Alt*A3R04 and *Mar*F3R11 reached 30%–33% of the final density in the formulated medium compared to the actual EPS from *Vib*1A01 (Fig 5F). Our mimicry medium likely underestimates the role of proteins and especially polysaccharides in promoting growth, primarily due to incomplete monosaccharide identification and limitations in distinguishing between unmodified and functionalized (e.g., acylated) polysaccharides with higher carbon content. Nevertheless, our results demonstrate that proteins, polysaccharides, and unidentified EPS components all serve as important cross-fed nutrient sources in chitin-degrading communities.

## Discussion

In this study, we demonstrate the critical role of EPS in mediating carbon flux and shaping microbial populations. Quantitative measurements of the exudate of the model chitin degrader *Vib*1A01 [6,9,31] reveal that EPS accounts for roughly one-quarter of the total carbon released—the remainder being low LMW metabolites. EPS yields were also consistent with those observed in other isolates and seawater communities. This substantial contribution underscores the potential of EPS as a major substrate in microbial crossfeeding. Our data further show that EPS is not a universally utilizable resource. EPS originating from the different chitin degraders selected for different communities when fed to natural seawater communities, enriching for specific EPS degraders. Among 18 tested isolates, only two exhibited robust growth on purified EPS, highlighting that EPS degradation is a specialized trait. Thus, the presence of abundant EPS in microbial ecosystems imposes a selective pressure that favors these specialized taxa.

Our results further show that EPS is degraded sequentially—first into larger oligomers, then into smaller mono- and oligomers. This process not only selects EPS-degrading specialists but also supports metabolically diverse non-degraders by generating common, accessible metabolite pools over time. Given EPS’s typical complexity as a mixture of multiple polymers, its breakdown likely produces a broad range of oligomers and monomers, which can sustain a diverse set of metabolically distinct microbes. We identified degradation products showing that EPS proteins and polysaccharides significantly support microbial growth and can be sequentially broken into amino acids and monosaccharides. However, other EPS components not resolved in this study likely contribute to carbon exchange. These may include humic-like substances with undefined structures and non-hydrolysable polysaccharides, such as acylated deoxy sugars, which are chemically recalcitrant and difficult to quantify. These compounds may represent a significant fraction of EPS and contribute to marine dissolved organic matter [45]. Ongoing research aims to characterize their composition and clarify their roles in marine carbon cycling. Our findings highlight the need to understand EPS composition both for its role in carbon flux and its influence on microbial community structure and function.

These findings advance our understanding of how metabolic cross-feeding shapes microbial community dynamics by highlighting the critical role of EPS. While most studies have focused on LMW compounds—central to known cross-feeding networks and foundational to many generalizable ecological principles [3,6,7,9]—we show that EPS contributes a comparable fraction of cross-fed carbon (about one-quarter in our case). Its sequential degradation by specialized subpopulations creates temporally dynamic metabolite pools that support metabolically diverse species. Given that microbial communities, particularly those degrading polysaccharides, often follow a temporal succession [26], this suggests that EPS production and degradation can influence community trajectories and help explain the coexistence of diverse taxa on a single carbon source. These insights are broadly applicable across ecosystems, as EPS is ubiquitous—produced as proteins, polysaccharides, and biofilms—and reveal an underappreciated layer of complexity that must be considered when linking cross-feeding to community structure and function.

## Methods

### Materials and chemicals

All chemicals, unless otherwise specified, were purchased from Sigma-Aldrich. The media used include Marine Broth 2216 (Thermo Fisher Scientific, Difco, no. 279100) and MBL minimal medium.

MBL medium consists of 1 mM phosphate dibasic, 1 mM sodium sulfate, and 50 mM TES (pH 8.2), along with three additional diluted stock solutions. Fourfold concentrated seawater salts (NaCl, 80 g/L; MgCl_2_*6H_2_O, 12 g/L; CaCl_2_*2H_2_O, 0.6 g/L; KCl, 2 g/L). 1000-fold concentrated trace minerals (FeSO_4_*7H_2_O, 2.1 g/L; H_3_BO_3_, 30 mg/L; MnCl_2_*4H_2_O, 100 mg/L; CoCl_2_*6H_2_O, 190 mg/L; NiCl_2_*6H_2_O, 24 mg/L; CuCl_2_*2H_2_O, 2 mg/L; ZnSO_4_*7H_2_O, 144 mg/L; Na_2_MoO_4_*2H_2_O, 36 mg/L; NaVO_3_, 25 mg/L; NaWO_4_*2H_2_O, 25 mg/L; Na_2_SeO_3_*5H_2_O, 6 mg/L, dissolved in 20 mM HCl). 1000-fold concentrated vitamins (riboflavin, 100 mg/L; d-biotin, 30 mg/L; thiamine hydrochloride, 100 mg/L; l-ascorbic acid, 100 mg/L; Ca d-pantothenate, 100 mg/L; folate, 100 mg/L; nicotinate, 100 mg/L; 4-aminobenzoic acid, 100 mg/L; pyridoxine HCl, 100 mg/L; lipoic acid, 100 mg/L; nicotinamide adenine dinucleotide (NAD), 100 mg/L; thiamine pyrophosphate, 100 mg/L; cyanocobalamin, 10 mg/L, dissolved in 10 mM MOPS, pH 7.2).

### Preparation of colloidal chitin

Ten grams of powdered chitin (Sigma-Aldrich, C7170) was dissolved in 100 mL of concentrated phosphoric acid (85% (w/v)) and incubated at 4 °C for 48 hrs. Approximately 500 mL of deionized water was added to this mixture and shaken vigorously until all the chitin precipitated. The precipitate was filtered using regenerated cellulose paper (MACHEREY-NAGEL, MN615). The chitin precipitate was then placed in cellulose dialysis tubing (approximately 13 kDa, Sigma-Aldrich D9652-100FT) and dialyzed with fresh deionized water daily for 3 days to remove residual phosphoric acid and oligomers. After dialysis, the pH was adjusted to 7 with 1M NaOH and homogenized using a Bosch SilentMixx Pro blender. The colloidal chitin was sterilized by autoclaving.

### Bacterial strains and seawater sampling

All bacterial species were stored in glycerol stocks at −80 °C. Before use, they were streaked onto Marine Broth 2216 plates with 1.5% (w/v) agar (BD, no. 214010) and incubated at room temperature until colonies formed. Overnight precultures were prepared by inoculating a single colony into 2 mL of Marine Broth 2216 and incubating in a 27 °C shaker overnight. In the case of *Psy*6C06, which grows to a low optical density in MB 2216 (OD_600_ ~ 0.2), the 2 mL preculture was used to inoculate a second overnight preculture of 15 mL.

Seawater was collected from Bogliasco, Italy (44.377477, 9.070292), and filtered through a 5-µM filter to obtain the bacterial fraction. Samples were aliquoted in 30% (v/v) glycerol and stored at −80 °C for future use as inoculum.

### pH-controlled bioreactor

A single pH-controlled bioreactor was set up in a custom-built incubator at 27 °C. The reactor was a 500 mL Erlenmeyer flask with 100 mL of MBL containing 2 g/L colloidal chitin and 50 mM sodium bicarbonate instead of TES buffer. As the bicarbonate equilibrated with the environment and increased the pH, 0.2 M sulfuric acid was titrated to pH 7.8 once the culture reached pH 8.1. The acid was in a separate bottle with a pierceable rubber cap (Fisher, 15896921). A feed tube transported acid between the feed bottle and the Erlenmeyer flask using a peristaltic pump (Ismatec). A stainless steel needle (18 gauge, 6-inch stainless steel 304 syringe needle, Sigma Z102717) withdrew medium from the feed bottle. This was connected to PharMed BPT tubing (2.79 mm) through a male Luer fitting for 1/8-inch tubing (Sigma 21016). This tubing was connected to PharMed 2-stop tubing (0.25 mm, Ismatec 95723-12) using another 1/8-inch male Luer fitting and a 22 gauge, 51-mm metal hub needle (Hamilton HAM191022) inserted into the 2-stop tubing. This 2-stop tubing was placed through the peristaltic pump and connected to an additional length of 2.79 mm PharMed BPT tubing and another 18 gauge stainless steel needle. This needle dispensed acid directly into the bioreactor. The pH was continuously monitored by a controller (Milwaukee PRO pH Controller MC-122) that regulated the power supply to the peristaltic pump.

The species were inoculated into the reactor at a cell concentration of 1 × 10^7^ cells/mL. Once the culture reached early stationary phase (no OD_600_ increase between consecutive time points), the culture was centrifuged to collect biomass, then sterile filtered through a 0.2-µM membrane. EPS and the LMW fraction were collected as described below. Before analysis, 100 mM HCl was added to each fraction to remove residual bicarbonate. Since there is no organic buffer in the medium, we can attribute measured carbon to be biologically derived. Samples of EPS (dialyzed in water), the LMW fraction (cell broth filtered through a 3 kDa membrane), and biomass (cell pellet), were freeze dried, weighed, and homogenized. Elemental analysis (Molecular and Biomolecular Analysis Service MoBiAS at ETH) quantified total carbon in these fractions, reporting the final concentration in percent carbon by weight. The presence of chitin was ruled out by monitoring chitin concentrations in the EPS and biomass fractions.

### Measuring EPS yields on chitin

30 mL cultures of 2 g/L chitin were inoculated with chitin-degrading isolates or frozen seawater stocks. Bacterial isolates were inoculated at a cell concentration of 1 × 10^7^ cells/mL. Seawater stocks were inoculated at a 1:100 ratio. Growth was monitored until early stationary phase, and the culture was centrifuged to collect biomass and sterile filtered through a 0.2-µM membrane. EPS and LMW fractions were collected as described below. To prevent suspended chitin from interfering with OD_600_ measurements, the sample was centrifuged for 30 s at 1,000 rcf, and the optical density of 100 µL of the supernatant was measured.

### Purification of EPS

Culture supernatant was fractionated into EPS and LMW fractions using a 3 kDa cutoff filter. For large-scale purification of 100 mL or more, an Amicon stirred cell was used to concentrate the medium down to less than 20 mL, where the concentrate was placed in a 1 kDa dialysis membrane and dialyzed in water. For 30 mL volumes, Amicon 3 kDa 15 mL centrifugal units were used, and rather than dialyzing, the concentrate was resuspended in water and further concentrated to dilute out salts three times. All filter units and MWCO (molecular weight cutoff) filters were washed by passing water through the membrane before use: 3 mL for the centrifugal units and 20 mL for the stirred cells. The resulting salt free, EPS concentrate was then freeze-dried and stored at −20 °C until further use. The LMW fraction is the filtrate of the 3 kDa filter.

### Growth of non-sterilized seawater on EPS

A preculture was prepared by inoculating frozen seawater 1:100 into MBL medium supplemented with 1 mM each of pyruvate, glutamate, glucose, acetate, succinate, and ammonium chloride. After 48 h of growth (OD₆₀₀ > 1), the culture was centrifuged and resuspended in fresh MBL without a carbon source. This was then inoculated 1:100 into 3 mL MBL containing 1 g/L EPS—the EPS harvested to quantify yields from isolates and seawater communities. After 48 h, growth was assessed, and cells were harvested for 16S rRNA sequencing.

### Growth of microbial isolates on EPS

Individual isolates were grown in 200 µL MBL containing 1 g/L EPS derived from *Vib*1A01, inoculated 1:100 from overnight cultures in MB. This EPS was harvested from a 100 mL *Vib*1A01 culture grown on chitin and concentrated using an Amicon stirred cell, as described above. Plates were incubated at 27 °C and shaken for 30 s, 2–3 times per day. OD₆₀₀ was measured periodically, and peripheral wells were filled with water to reduce evaporation.

### Excitation Emission measurements and PARAFAC

Emission excitation matrices were acquired using a Tecan M plex plate reader, with excitation wavelengths between 250 and 450, and emission wavelengths between 300 and 600, measured at 5 nm intervals. Coble peak integration, PARAFAC models, and component integration was performed using staRdom [46] R package.

### 16S sequencing of microbial communities grown on EPS

Frozen pellets containing 1 OD-mL equivalent were used for genomic DNA extraction with the Monarch Genomic DNA Purification Kit (New England Biolab). DNA quantity and quality were then assessed using a qubit fluorometer and a nanodrop spectrophotometer and all samples were diluted to 3.33 ng/µL. For each sample, 10 ng (3 µL) of genomic DNA was used to prepare sequencing libraries using the 16S Barcoding Kit 24 v14 kit (Oxford Nanopore) following manufacturer’s instructions with a unique barcode for each sample. After library preparation, samples were pooled and sequenced on a MinION Mk1B sequencer with a Flongle adapter using R10 flongle flow cells. Reads were filtered for size and quality using Cutadapt [47], yielding an average of ~30,000 filtered reads per sample. The filtered reads were then used to estimate species relative abundances using Emu [48] with default parameters.

### Protein measurements

Proteins were quantified by adding 30 µL of sample to 300 µL of Bradford reagent (Thermo Scientific Pierce Bradford Protein Assay Kit) and quantification used standards of bovine serum albumin.

### EPS digestion using enzyme cocktails

Here, we adapted a protocol based on a previous approach using microbial enzyme cocktails to degrade and characterize polysaccharides [49]. Three milliliters of EPS-containing medium (1 g/L in MBL with 1 mM ammonium chloride) were inoculated with seawater and grown at 27 °C for 72 h until saturation. Cells were centrifuged, and the supernatant was sterile filtered using a 0.2-µM filter. Enzymes were concentrated 10-fold using a 10 kDa Amicon centrifugal unit, with excess salts and small molecules removed by adding water and reconcentrating, repeated three times. This procedure was done in parallel for EPS substrates from each of the three degraders, yielding three sets of enzyme cocktails. The enzyme assay included 10 mM ammonium bicarbonate pH 7.8, 1 g/L EPS from one degrader, and the enzyme cocktail. The reaction was initiated by adding 50 µL of enzyme to 120 µL of the other components and was performed in parallel with a no-enzyme control to account for non-enzymatic hydrolysis of EPS. The reaction was performed with four replicates, where degradation products in three replicates were monitored in real time using LC–QTOF–MS for 24 h. The fourth replicate was frozen immediately after the 24-h incubation and later analyzed to acquire MS/MS spectra for compound identification.

### LC–MS measurements

The enzyme assay was initiated in the autosampler of an Agilent Infinity II 1290 multisampler and continuously sampled for 24 h. The LC–MS method used an Agilent Poroshell PFP column (50 × 2.7 mm, 1.9 µM) at 30 °C. Mobile phase A was 100% water with 0.1% formic acid (v/v), and mobile phase B was 10% acetonitrile with 0.1% formic acid (v/v). The method started with 100% phase A for 1 min, decreased to 70% A over 30 s, then to 10% A over another 30 s, held for 30 s, and returned to 100% A for 3 min of equilibration before the next injection. An Agilent 6,546 Q-TOF mass spectrometer measured product formation, with each sample injected in negative mode, followed by positive mode. Samples were acquired in high-resolution mode, 50–1,700 *m/z* range, 6 Hz scan speed, fragmentor 110 V, drying gas 10 L/min, and capillary voltage of 3,500 V.

Peak detection and integration were performed using MZmine version 3.4.1 [50]. Ion features were aligned across all samples, and their intensities were compared over time to identify ions with changing concentrations. Specifically, ions that exhibited an increase over time—defined as those with a log₂ fold change in intensity >10 when comparing the 24-h time point to the initial time point in one of the enzyme digests—were selected for further analysis. An inclusion list of these increasing ions was generated to acquire MS^2^ spectra for compound class identification. The same LC–MS method was employed as previously described. MS^2^ spectra were acquired using collision-induced dissociation with a collision energy of 25 V. Data acquisition was performed with a dwell time of 50 ms, a cycle time of 500 ms, and a ~ 1.3 *m/z* isolation window.

### Mock *Vib*1A01 EPS medium

Medium mimicking the proteins and polysaccharides present in the medium containing 1 g/L *Vib*1A01 EPS contains galactose (13 µM), galacturonic acid (17 µM), glucosamine (22 µM), glucose (20 µM), and 150 mg/L bovine serum albumin. Microbial isolates were grown in 200 µL of this medium alongside 200 µL of 1 g/L *Vib*1A01 EPS.

### Materials and methods NMR

Samples of 20 mg EPS have been dissolved in 150 uL D_2_O (cat. number 756822). The reference samples were 250 mM GlcNAC (Sigma) and mM chitobiose (Omicron Biochemicals cat. Number DIS-013) in 150 μL D_2_O. The solutions were transferred to 3 mm NMR tubes (Norell, S-3-HT-7), and NMR data were acquired at 303.0 K at a 600 MHz Bruker AVNEO NMR spectrometer equipped with a CP-TCI-H-C/N-D 05 Z probe head.

1D ^1^H experiments were acquired with 8,192 complex data points and 32 transients. The remaining solvent signal was suppressed by applying presaturation or the watergate sequence with soft selective pulses [51].

2D ^1^H-^13^C HSQC experiments were acquired with 2048 complex data points in the direct dimension, 16 transients, and 320 data points in the indirect dimension. For data acquisition and analysis, the program Topsin4.0.7 (Bruker Biospin) was used. Data were multiplied with a squared Cosine bell window function and zero-filled to two times the original data size. Data are stored in S11 Data.

### Monosaccharide analysis of EPS

EPS of bacterial supernatants were acid hydrolyzed with 1 M HCl at 100 °C for 24 h. Additionally, each sample was spiked with an internal standard of 15 µM 13C6-glucose, 13C6-galactose, and 13C6-mannose (mass 186 Da). Samples were neutralized with equimolar amounts of NaOH. Released monosaccharides (5 µL) were diluted with 20 µL of LC–MS grade water and subsequently derivatized with 75 µL of 0.1M 1-phenyl-3-methyl-5-pyrazolone (PMP) in 2:1 methanol:ddH_2_O with 0.4% ammonium hydroxide, following a previously published protocols [52]. For quantification, we derivatized a serial dilution of a standard mix containing galacturonic acid, D-glucuronic acid, mannuronic acid, guluronic acid, xylose, arabinose, D-glucosamine, fucose, glucose, galactose, mannose, N-acetyl-D-glucosamine, N-acetyl-D-galactosamine, N-acetyl-D-mannosamine, ribose, rhamnose, and D-galactosamine. Samples and standards were derivatized by incubation at 70 °C for 100 min. After derivatization, samples were neutralized with HCl followed by 1:50 dilution in LC–MS grade water with 0.1% formic acid.

Following Xu and colleagues [53], PMP derivatives were measured on a SCIEX qTRAP5500 and an Agilent 1290 Infinity II LC system equipped with a Waters CORTECS UPLC C18 Column, 90 Å, 1.6 µm, 2.1 × 50 mm reversed phase column with guard column. The mobile phase consisted of buffer A (10 mM NH_4_ formate in ddH_2_O, 0.1% formic acid) and buffer B (100% acetonitrile, 0.1% formic acid). PMP derivatives were separated with an initial isocratic flow of 15% buffer B for 2 min, followed by a gradient from 15% to 20% buffer B over 5 min at a constant flow rate of 0.5 mL /min and a column temperature of 50 °C. The ESI source settings were 625 °C, with curtain gas set to 30 (arbitrary units), collision gas to medium, ion spray voltage 5,500 (arbitrary units), temperature to 625 °C, ion source gas 1 and 2–90 (arbitrary units). PMP derivatives were measured by multiple reaction monitoring (MRM) in positive mode with previously optimized transitions and collision energies. Different PMP derivatives were identified by their mass and retention in comparison to known standards. Technical variations in sample processing were normalized by the amount of internal standard in each sample. Peak areas of the 175 Da fragment were used for quantification using an external standard ranging from 100 pM to 10 µM.

### Chitin quantification

To quantify chitin remaining after degraders were grown in chitin cultures, we followed our previously published chitin quantification protocol [49] using concentrated enzyme cocktails from *Psy*6C06. *Psy*6C06 was inoculated at 1% from an MB2216 preculture into 400 mL of MBL medium with 2 g/L colloidal chitin and shaken at 200 rpm until early stationary phase. After growth, the medium was centrifuged at 2,800 rcf for 20 min to remove any remaining chitin and cells, then sterile filtered using a 0.2-µm membrane. This supernatant was concentrated 10-fold using an Amicon stirred cell (Millipore) with a 3 kDa cutoff filter. A protease inhibitor (Roche cOmplete EDTA-free protease inhibitor cocktail) was added to the final concentrate. 500 µL aliquots were placed in 1.5 mL microcentrifuge tubes, snap-frozen in liquid nitrogen, and stored at −80 °C until use.

Frozen enzyme aliquots were thawed on ice. 100 µL of enzyme was added to 100 µL of bacterial freeze-dried biomass and incubated at room temperature for 72 h. The assay was centrifuged at 2,800 rcf for 1 min, and the supernatant was collected and monomers were quantified using a DNS-reducing sugar assay. A calibration curve of samples containing known concentrations of colloidal chitin were simultaneously digested to derive quantitative concentrations in the biomass samples.

To prepare the DNS reagent, 100 mL of water was heated to 70–75 °C, then poured over 2 g of NaOH pellets with constant stirring. 2.18 g of 3,5-dinitrosalicylic acid was dissolved in this mixture, followed by 30 g of sodium potassium tartrate (Rochelle salts). The solution was cooled to room temperature and stored in the dark. For the assay, 25 µL of sample was added to 75 µL of DNS reagent and heated to 95 °C for 15 min in a thermocycler. Eighty µL was transferred to a 384-well plate, and a colorimetric readout was measured at OD540.

### DNA quantification

EPS was added to water at a concentration of 1 g/L and sonicated for 20 min to break apart any insoluble particles. DNA was quantified using a Qubit 4 fluorometer with a dsDNA BR Assay Kit (Thermo), where 5 µL of 1 g/L EPS was used as an input for measurements.

### MS^2^-based compound class annotations

For MS^2^-based annotation, fragmentation spectra were extracted using MzMine (version 4.3.0) [50]. Parameters used for mass detection were a minimal intensity of 500 for MS^1^ and 100 for MS^2^. The feature list was built using the MS^n^ tree builder with a tolerance of 20 ppm. Only features with associated MS^2^ were kept. Isotopes were searched within a 3 ppm tolerance window, features lists were aligned using a 0.1 min and 8 ppm tolerance window. Finally, the feature list was deduplicated using a 20 ppm and 0.1 min window and exported for later use in SIRIUS (with a 5 ppm tolerance).

Extracted spectra were then submitted to SIRIUS [54] v 6.0.7 and CANOPUS [44] module for compound class annotation. All parameters used were the software default ones.

The obtained annotations were then filtered using a custom R script that combined the SIRIUS results tables together and only kept results with at least 0.5 intensity explained, an isotope score of at least 5 if present, and a most specific class probability above or equal to 0.75. The resulting table is available as S9 Data.

## Supporting information

S1 Data All raw data for Fig 1. (XLSX)

S2 Data All raw data for Fig 2A, 2B, 2D, and 2E. (XLSX)

S3 Data All raw data for Fig 2C. (XLSX)

S4 Data All raw data for Fig 3. (XLSX)

S5 Data All raw data for Fig 4A and 4C. (XLSX)

S6 Data Raw metabolomics data for all ions that showed a detectable increase in concentration at any time point compared to time point 0 in the EPS enzyme digests. (XLSX)

S7 Data All raw metabolomics data for Figs 4B and 5B. (XLSX)

S8 Data Annotated ions found in the EPS enzyme digests. (XLSX)

S9 Data Compound classes identified based on MS^2^ data for ions found in the enzyme digests. (XLSX)

S10 Data All raw data for Fig 5A, 5C, 5D, and 5F. (XLSX)

S11 Data Raw data for NMR in Fig 5E. (ZIP)

S1 Table All monosaccharide concentrations in each EPS sample, extended from Fig 2B. (CSV)

## Abbreviations

EPS: extracellular polymeric substance
LC–QTOF–MS: liquid chromatography quadrupole time of flight mass spectrometry
LMW: low-molecular-weight
MRM: multiple reaction monitoring
NAD: nicotinamide adenine dinucleotide
PMP: phenyl-3-methyl-5-pyrazolone
